# Reasons to quit cigarettes, e-cigarettes, and smokeless nicotine products: A cross-sectional study among Danish people aged 15–29 years

**DOI:** 10.18332/tpc/215586

**Published:** 2026-05-22

**Authors:** Stine Arp, Christina M. Stapelfeldt, Lotus S. Bast

**Affiliations:** 1 National Institute of Public Health, University of Southern Denmark, Copenhagen, Denmark; 2 Institute of Public Health, Aarhus University, Aarhus, Denmark; 3 The Danish Healthcare Quality Institute, Aarhus, Denmark

**Keywords:** motivation, nicotine, cigarettes, e-cigarettes, nicotine cessation

## Abstract

**INTRODUCTION:**

Following the increased use of e-cigarettes and smokeless nicotine products among Danish youth, the proportion of youth wishing to quit or already had attempted to quit has increased too. To adapt cessation services, knowledge is needed about youth self-reported reasons to quit and whether reasons differ across nicotine products.

**METHODS:**

In a cross-sectional study carried out from September to November 2023, using age and gender-weighted questionnaire data, 2228 Danish youth aged 15–29 years, that had attempted to quit one or more nicotine product (cigarettes, e-cigarettes or smokeless nicotine) were included. Differences in self-reported reasons across products used were assessed using unadjusted logistic regression. A supplementary interaction analysis, using quit success as the interacting variable, and unadjusted logistic regressions stratified by quit success, were performed to assess the role of quit success on the most reported reasons.

**RESULTS:**

‘Thoughts about health’ and ‘Don’t want to be addicted’ were the most frequently reported reasons to quit cigarettes (51.5% and 39.1%, respectively), e-cigarettes (38.8% and 33.6%, respectively) and smokeless nicotine (61.3% and 60.3%, respectively). However, prevalences varied between products. ‘Due to economics’ was the third most prevalent reason among youth who had attempted to quit cigarettes (29.0%) and smokeless nicotine (40.7%), whereas this was ‘Switching to another product’ among those who had attempted to quit e-cigarettes (21.5%). Quit success significantly modified the reporting of ‘People I know privately’ as reason for quit attempt, across the products.

**CONCLUSIONS:**

Counselors, parents, and those developing new interventions should consider health and addiction concerns as reasons for wanting to quit among youth, and must be aware of different reasons for quitting different products. Further studies are needed to verify the present findings and their interpretation related to intrapersonal, interpersonal and structural issues.

## INTRODUCTION

As the smoking prevalence among youth in Denmark stagnated, the use of alternative nicotine products, such as electronic cigarettes (e-cigarettes) and snus, chewing tobacco and nicotine pouches (smokeless nicotine) had rapidly gained popularity among Danish youth over the past decade^1^. For example, e-cigarette use among Danish youth rose from 3.9% in 2020 to 10.4% in 2024. And among participants aged 15–17 years, the use tripled in the same period from 4.5% to 14.3 %. An increase that can largely be attributed to the rapidly increasing use of disposable e-cigarettes. Furthermore, use of smokeless nicotine similarly increased from 9.1% to 12.6% from 2020 to 2024, and most prominently among those aged 18–24 years from 11.9% to 16.2%^1^. Despite limited studies on the long-term health effects of smokeless nicotine, various studies found that nicotine exposure in adolescence induces damage to the developing brain^2-4^ and the cardiovascular system^5,6^. Smokeless tobacco (Swedish snus) has been found to contain carcinogens and therefore entails a potential cancer risk^7^. Similarly, use of e-cigarettes, specifically, has been found to increase the risk of respiratory diseases^8,9^. Lastly, nicotine’s high addictiveness poses a risk of lifelong addiction^2,10^ and potentially serves as a gateway to other harmful substances^4,11^.

As the use increased, so did the number of youths considering quitting. In 2024, a total of 47.4% of youth who used e-cigarettes had considered quitting in the past month, while 25.3% had attempted to quit in the past year^1^. Furthermore, 72.3% had considered quitting smokeless nicotine in the past month and 48.1% had been making a past-year quit attempt^1^. International evidence on smoking cessation indicates that less than 10% of youth who attempt to quit unassisted remain abstinent^12,13^. Conversely, based on a national database, 37% achieved a six-month abstinence from cigarette smoking, 56% from e-cigarettes and 64% from smokeless nicotine, among Danish youth up to 35 years old, attending quit cessation services^14^. Yet, we do not know whether they succeeded in quitting nicotine completely or if they instead switched to another product. Also, participation in cessation services was markedly lower among youth using e-cigarettes (1%) and smokeless nicotine (3%), than youth using cigarettes (91%)^14^, and the free of charge cessation services in Denmark are experiencing difficulty in recruitment, counseling, and retention of youth below the age of 25 years, compared to adults^15^.

Prior studies found health concerns^16-24^, costs^21-23,25-28^, and relationships^21-24,26,28^ as prevalent reasons for quitting both cigarettes and e-cigarettes. However, image and aesthetics were more frequently mentioned as quitting reasons among youth using cigarettes^16,19,24,27,28^, whereas addiction was mentioned more frequently among youth using e-cigarettes^20-24,26,27^. Research regarding reasons to quit smokeless nicotine is, however, limited. A qualitative study among participants aged 18–25 years in the US, presented pressure from network and health concerns as motivating reasons for smokeless tobacco cessation^24^. Also, a recent survey involving Danish municipalities, found that the three most motivating reasons to quit nicotine among Danish youth were costs, addiction, and parental pressure^15^. However, this was reported by counselors and type of nicotine product was not specified.

Vu et al.^24^ found that American youth reported taste, easy access, and low cost as reasons to initiate e-cigarette and smokeless tobacco use more often compared to cigarette initiation, while Berg et al.^29^ reported that motives for initiating vaping may include perceptions of e-cigarettes being less harmful than cigarettes and could be used where smoking was prohibited. Such differences in initiation motives across products may indicate similar trends regarding reasons to quit. Further, as prior studies suggest that health and social reasons were more commonly reported among successful cigarettes quitters^30^, we further hypothesized that reported quit reasons differ according to success of prior quit attempt.

Understanding the reasons why youth attempt to quit different nicotine products is essential for cessation services and those developing preventive interventions for youth nicotine cessation. Therefore, this study aims to examine self-reported reasons for prior quit attempt among Danish youth and to explore whether these reasons vary depending on the product used. Additionally, to understand whether quit success influences the reporting of certain reasons across products, we further aim to investigate if the association between product and reason is modified by quit success.

## METHODS

### Study design

This study uses cross-sectional data from the fifth survey of the Danish nationwide evaluation, §SMOKE – A study of tobacco, behavior, and regulations. Briefly, §SMOKE consists of annually repeated cross-sectional surveys from 2020 to 2025, including youth aged 15–29 years randomly recruited through the Danish Civil Registration System. §SMOKE is carried out by the National Institute of Public Health in collaboration with The Danish Cancer Society, The Heart Association and The Lung Association^31^.

The fifth survey was collected from September 2023 to November 2023. Questionnaires were distributed to a random sample of Danish citizens (n=37586) aged 15–29 years through Digital post, a mandatory and secure mailing system. Answers were obtained from 9264 (24.6%) respondents, and 2228 were included in the analyses after excluding invalid responses (n=2), respondents who had never used any nicotine product (n=4063) or were missing according to product use (n=2015), respondents who had used nicotine products, but had never attempted to quit (n=765), or were missing according to quit attempt (n=120). Also, youth who had attempted to quit, but had not reported reasons were excluded (n=71). Briefly, reporting reasons were missing for 3.3% (n=56) who had attempted to quit cigarettes, 1.5% (n=9) who had attempted to quit e-cigarettes, and 2.1% (n=16) who had attempted to quit smokeless nicotine.

### Measures

*Nicotine product use*

Cigarette use was assessed by asking: ‘Do you smoke cigarettes?’. Responses ‘Yes, every day’, ‘Yes, at least once a week’ or ‘Yes, less often than every week’ indicated current use. Responses ‘No, but I have tried’ or ‘No, I have never tried’ indicated no current use. Whereas ‘No, but I have been using in the past’ indicated former use. E-cigarette and smokeless nicotine use were coded similarly.

*Quit attempt*

Youth who had attempted to quit included: 1) those with a current use, who reported ever attempting to quit; and 2) those with a former use. Youth currently using cigarettes, e-cigarettes and smokeless nicotine were asked: ‘Have you attempted to quit using cigarettes/e-cigarettes/smokeless nicotine in the following period?’. If answered ‘Yes, in the past 12 months’ and ‘Yes, for more than a year ago’ respondents were regarded as having ever attempted to quit.

*Reasons to quit*

Respondents who had attempted to quit were posed the following question for each product: ‘What prompted you to initiate your most recent quit attempt?’. Eleven predefined response items were: ‘Was encouraged by health personnel’, ‘Something I have seen or heard in the media’, ‘Pictures or text on the packaging’, ‘People whom I know privately’, ‘Due to economics’, ‘Due to regulations on tobacco and nicotine use’, ‘Thoughts about my health’, ‘Don’t want to be addicted’, ‘Switched to another product’, ‘Other’ and ‘Do not remember’. Each item was categorized as 1 if chosen and 0 if not. It was possible to answer in free text, which were manually recoded if covered by one or more of the predefined items.

*Quit success*

Among those who had attempted to quit a specific product, former use of that product indicated quit success, whereas current use indicated no quit success.

*Sociodemographic factors*

The respondents age at the time of filling in the survey, gender, and region, were retrieved through the Danish Civil Registration System. Age was subsequently grouped into those aged 15–17 years, 18–24 years, and 25–29 years. Occupation status was coded based on self-reported current working status and educational status, resulting in 11 categories.

### Statistical analysis

Data were reorganized so that the number of individual respondents (n=2228) to the number of respondents who attempted to quit one single product (n=2948). Thus, a respondent who had attempted to quit two products, counted as two observations, one per product. Descriptive statistics were calculated as percentages, that were weighted according to the age and gender distribution in the background population. Eleven unadjusted logistic regressions with product as the exposure and the reason under consideration as the outcome were made, followed by a Wald chi-squared test testing for overall difference across the three products. As establishing causal directions were not the aim of this study, and unfeasible due to the cross-sectional design, we did not adjust for confounders. Violation of the independence assumption was addressed by using cluster-robust standard errors. To improve representability, data were weighted for non-response according to gender and age in the randomly invited sample. Interaction between product type and quit success on the five most frequently reported reasons was addressed using unadjusted logistic regression and were subsequently stratified by quit success. Interaction between product and quit success and overall difference across the three products in the two strata was tested by a Wald chi-squared test. Analyses were carried out by Stata version 18 and p<0.05 was considered significant.

## RESULTS

### Description of population

Table 1 presents characteristics of the total sample and included sample, divided by product used. Most of the included respondents have attempted to quit cigarettes (55.9%). The prevalence of attempting to quit smokeless nicotine was higher among males (63.4%) than females (36.6%). Gender was somewhat equally distributed for those attempting to quit cigarettes and e-cigarettes, and in the total sample. Youth who attempted to quit e-cigarettes fairly resembled the total population according to age, whereas attempting to quit cigarettes was more prevalent among those aged 25–29 years (51.8%), and attempting to quit smokeless nicotine was more prevalent among participants aged 18–24 years (60.1%). Youth who attempted to quit cigarettes were almost twice as likely to be working, with a medium- or long-cycle higher education, than youth who had attempted to quit e-cigarettes and smokeless nicotine. However, youth who had attempted to quit e-cigarettes and smokeless nicotine were more likely to attend upper secondary education. Quit success was more prevalent among youth who had ever attempted to quit e-cigarettes (79.6%) and least prevalent among those who had ever attempted to quit smokeless nicotine (36.4%). Lastly, those who attempted to quit e-cigarettes reported fewer reasons than those who had attempted to quit the other products.

**Table 1:** Sample characteristics^a^ of total sample (N=9262) and the included sample (N=2948), Denmark, 2023

*Characteristics*	*Total sample*		*Ever quit attempt (N=2948)^b^*					
			*Cigarettes*		*E-cigarettes*		*Smokeless nicotine*	
	*%*	*n*	*%*	*n*	*%*	*n*	*%*	*n*
**Total**	100	9262	55.9	1620	19.2	589	24.9	739
**Gender**								
Male	51.0	4002	51.7	703	51.3	249	63.4	408
Female	49.0	5260	48.3	917	48.7	340	36.6	331
**Age** (years)								
15–17	18.4	2029	7.7	159	16.0	117	10.7	98
18–24	45.4	4467	40.5	734	49.7	313	60.1	473
25–29	36.2	2766	51.8	727	34.3	159	29.2	168
**Region**								
Northern Denmark	10.2	942	8.9	139	9.6	55	11.5	87
Central Denmark	25.8	2391	23.7	380	22.1	129	27.1	197
Southern Denmark	21.1	1954	19.5	314	21.5	124	19.6	149
Capital	31.7	2908	35.3	573	32.9	196	31.6	227
Zealand	11.2	1067	12.6	214	13.9	85	10.2	79
**Occupation**								
Attending lower secondary education	6.7	723	2.0	40	4.0	29	1.7	15
Attending preparatory basic education or vocational education	5.9	547	7.7	130	10.7	63	6.2	45
Attending upper secondary and vocational education	1.5	148	0.9	17	2.2	14	2.3	19
Attending upper secondary education	16.6	1735	8.7	165	14.4	100	14.6	125
Attending a short-cycle higher education	2.0	178	2.0	34	1.8	10	2.1	16
Attending a medium- or long-cycle higher education	20.9	1907	20.0	326	15.2	87	27.5	198
Attending single-subject courses/other	3.1	296	2.7	48	2.3	14	1.7	14
Working, with a short-cycle higher education or less	23.1	2025	31.9	493	31.9	179	30.3	218
Working, with a medium- or long-cycle higher education	14.5	1145	15.8	231	8.1	40	8.0	47
Not working, with a short-cycle higher education or less	4.5	406	6.6	109	8.6	49	4.8	37
Not working, with a medium- or long-cycle higher education	1.2	101	1.7	27	n/a		0.8	5
**Quit success**								
Unsuccessful (current use)			46.4	764	20.4	128	63.6	475
Successful (former use)			53.6	856	79.6	461	36.4	264
**Number of items reported, median** (IQR)			2	(1-3)	1	(1-2)	2	(1-3)

a Percentages, median, and IQR are weighted. Numbers are non-weighted;

b 2948 observations correspond to 2228 individuals, as some are using more than one product. IQR: interquartile range.

### Reasons to quit

Most of the youth who attempted to quit cigarettes (51.5%), e-cigarettes (38.8%), and smokeless nicotine (61.3%), reported ‘Thoughts about my health’ as a reason for their quit attempt (Table 2). However, the proportion of youth reporting this reason differed significantly across products. Thus, youth who had attempted to quit smokeless nicotine were more likely to report this as a reason for their quit attempt, than those who had attempted to quit cigarettes and e-cigarettes. ‘Don’t want to be addicted’ was the second most reported reason for all products. Similarly, this was reported by a significantly higher proportion of youth who had attempted to quit smokeless nicotine (60.3%), than those who had attempted to quit cigarettes (39.1%) and e-cigarettes (33.6%). The third most reported reason for attempting to quit cigarettes and smokeless nicotine was ‘Due to economics’ and was more frequently reported by youth who had attempted to quit smokeless nicotine (40.7%), than those who had attempted to quit cigarettes (29.0%) and e-cigarettes (16.9%). The third most reported reason to quit e-cigarettes was ‘Switching to another product’, which was more prevalent among youth who had attempted to quit e-cigarettes (21.5%) and cigarettes (19.1%) compared to those who had attempted to quit smokeless nicotine (4.4%).

**Table 2:** Reasons to quit among youth who attempted to quit cigarettes, e-cigarettes, and smokeless nicotine, Denmark, 2023 (N=2948)^b^

	Total		Cigarettes		E-cigarettes		Smokeless nicotine		p*
	%	n	%	n	%	n	%	n	
**Total^a^**	100	2948	100	1620	100	589	100	739	
**Contributing reason for recent quit attempt**									
Thoughts about my health	51.5	1505	51.5	822	38.8	233	61.3	450	**<0.001**
Don’t want to be addicted	43.3	1278	39.1	633	33.6	202	60.3	443	**<0.001**
Due to economics	29.6	876	29.0	473	16.9	100	40.7	303	**<0.001**
People I know privately	20.0	595	19.6	320	15.8	96	24.2	179	**<0.001**
Other	17.2	506	20.7	336	20.0	117	7.0	53	**<0.001**
Switched to another product	15.9	463	19.1	310	21.5	121	4.4	32	**<0.001**
Encouraged by healthcare professionels	4.6	132	4.5	70	3.9	23	5.5	39	0.37
Something I have seen or heard in the media	4.5	142	2.8	46	7.7	50	5.8	46	**<0.001**
Do not remember	4.3	132	3.2	53	8.6	51	3.6	28	**<0.001**
Due to regulations on tobacco and nicotine use	3.1	89	3.5	56	3.8	20	1.7	13	0.05
Pictures or text on the packaging	1.4	43	1.9	31	1.0	6	0.7	6	**0.03**

a Numbers are observations (individuals using the respective product) and are presented unweighted. Percentages are presented as weighted estimates.

b 2948 observations correspond to 2228 individuals, as some are using more than one product.

* Calculated by Wald tests using logistic regression with clustering on id; significant at p<0.05.

Quit success significantly modified the association between product type and the selection of ‘People I know privately’ as a reason for quitting (p=0.022) (Supplementary file Table 1). This was driven by differences among those attempting to quit e-cigarettes. Among unsuccessful users those who had tried to quit e-cigarettes had a tendency towards higher odds of choosing this reason compared to unsuccessful quitters of cigarettes (OR=1.38; 95% CI: 0.89–2.11). In contrast, among successful quitters, those who succeeded in quitting e-cigarettes had significantly lower odds of reporting this reason, compared to successful quitters of e-cigarettes (OR=0.66; 95% CI: 0.49–0.88).

## DISCUSSION

The present study found that ‘Thoughts about health’ and ‘Don’t want to be addicted’ were the most frequently reported reasons to quit all three nicotine products. However, the prevalence of the reported reasons generally differed across product type. ‘Due to economics’ remained the third most prevalent reason among youth who had attempted to quit cigarettes and smokeless nicotine, whereas for those who had attempted to quit e-cigarettes, the third most prevalent reason was ‘Switching to another product’. Further, interaction between product type and quit success was observed for reporting the reason ‘People I know privately’.

Health concern was the most frequently reported reason to quit across all nicotine products, in accordance with previous studies of cigarette and e-cigarette cessation among youth^16,20,23,26^. Although one study identified health as a less prominent reason^17^, differences in item definitions and study designs may explain this inconsistency. Similarly, qualitative studies highlighted health as an important reason in attempting to quit both cigarettes, e-cigarettes, and smokeless tobacco^18,19,21,22,24^. The additional finding that health reasons were reported more frequently among youth who had attempted to quit cigarettes compared to e-cigarettes, is similarly consistent with previous studies, when comparing the reported frequencies regarding reasons to quit cigarettes^16,17^ and e-cigarettes^20,23,24,26^. We also found that youth who had attempted to quit smokeless nicotine were more likely to report health reasons than those attempting to quit cigarettes and e-cigarettes. While comparative international data are limited, this finding contrasts with a qualitative study from 2022 among Danish youth, finding that youth generally attach less importance to the health risks of smokeless nicotine^32^. In 2022, mass communication regarding health effects of nicotine pouches intensified in Denmark^33^, which might have altered the health perception among youth and could possibly explain our findings. However, more research is still needed addressing youth’s perception of health consequences of smokeless nicotine in a cessation-setting.

Concerns about addiction were reported as the second most common reason to quit, and was particularly frequent among youth who attempted to quit smokeless nicotine. Given that smokeless nicotine products can contain higher levels of nicotine than cigarettes^34^, a higher risk of nicotine addiction among youth using smokeless nicotine could explain these findings. This also aligns with the fact, that more Danish youth using smokeless nicotine perceiving themselves as addicted, compared to those using cigarettes^1^. Youth who attempted to quit e-cigarettes were further less likely to report this reason than those attempting to quit cigarettes. This is contradictory to other studies, in which addiction was mentioned more frequently among youth attempting to quit e-cigarettes^20,23,26^ than those attempting to quit cigarettes^16^.

Economic reasons were more frequently reported by youth who had attempted to quit smokeless nicotine than those who had attempted to quit cigarettes. According to a former study, youth’s sensitivity to changes in cigarette price decreases with age^35^. As those who had attempted to quit smokeless nicotine were generally younger than those attempting to quit cigarettes, their lower age could explain these findings. This explanation cannot, however, be applied to youth who had attempted to quit e-cigarettes, as they were further less likely to report economic reasons than those attempting to quit cigarettes, even though they were younger. This difference is supported by previous studies of youth reporting economy as a reason to quit cigarettes^16,17^ and youth attempting to quit e-cigarettes^23^. This may suggest that economic factors might be less relevant among those attempting to quit e-cigarettes, potentially due to lack of price regulations like those for cigarettes. As price regulation is a widely used tobacco control strategy, further research is needed to clarify how pricing influences motivations to quit different nicotine products. Notably, since data for this study were collected, a tax increase on nicotine pouches has been implemented^36^. Future studies could therefore examine whether reporting economic reasons to quit smokeless nicotine change following such policy implementations.

Quit success modified the association between product type and reporting ‘People I know privately’ as a reason to quit. Briefly, this means that the lower odds of reporting this reason if the respondent had attempted to quit e-cigarettes (compared to cigarettes) were further reduced if the respondent had also successfully quit. Similarly, the stratified analysis showed that those who had successfully quit e-cigarettes reported this reason less frequently than those who had successfully quit cigarettes. Since social support has been found to predict successful quitting^30^, findings could indicate a stronger social support to quit among youth using cigarettes than those using e-cigarettes. Due to limited evidence about health risks of e-cigarettes and a perception that it is safer than cigarettes^24^, youth attempting to quit e-cigarettes might not experience the same level of positive social support as youth attempting to quit cigarettes.

In summary, we found that reasons to quit differed according to product used. This is valuable knowledge for the nationwide cessation services experiencing trouble with recruiting, counselling and retention of youth. A Danish survey including municipal counselors by Rasmussen and Pisinger^15^ found economy, addiction, and pressure from parents a the three most reported reasons to quit any nicotine products among youth. This is distinct from our findings, where health concerns were mentioned more frequently than economy and addiction. The results reported by Rasmussen and Pisinger^15^ are based on counselors’ experiences and this difference may emphasize a discrepancy between what the youth are experiencing as motivating and what the counselors perceive in their meetings with them. Further explanations could be that the youth that presented these perspectives among counselors, may represent a more motivated group, as they have already been in contact with the counselors.

### Strengths and limitations

This study has several strengths. Compared to other studies of youth attempting to quit nicotine products, this study included a relatively large sample size. Also, no study has to our knowledge shed light on the differences in reasons to quit across nicotine product types. In addition, including smokeless nicotine was a significant strength, given that the use is highly prevalent in Denmark^1,37^ and thus the need for knowledge about helping youth to quit is much needed.

This study also has some limitations. First, because of the cross-sectional design, it was not possible to indicate any causal direction between reason to quit and quit success. Second, the overall response rate was low. However, data were weighted to account for non-response, which diminishes selection bias arising from uneven response rates across age and gender. Selection bias due to other factors, i.e. socioeconomic status, could, however, be present. Third, few respondents did not report reasons to quit (n=71). However, as these responders are equally distributed across products, results were not affected. Fourth, youth who attempted to quit e-cigarettes reported ‘Do not remember’ more frequently. This may introduce information bias, due to differential misclassification of the outcome, dependent on the exposure, as an underestimation of the predefined reasons among those using e-cigarettes, may cause an overestimation of the differences in reported reasons between those using e-cigarettes and those using other products. Lastly, analyses were unadjusted, enhancing the risk of biased estimates, due to confounding factors, i.e. age and gender. However, age at the time of quit attempt was not measured, and adjusting for age at data collection would not be informative. Also, as causality was not the aim of this study, adjusting for potential confounding factors was not deemed relevant. It should further be noted that the findings may only apply in a Danish context, or contexts similar to this, although we found several agreements in findings with studies from other countries.

## CONCLUSIONS

This nationwide study of youth who attempted to quit cigarettes, e-cigarettes, and/or smokeless nicotine showed that reasons to quit differed according to product used. However, concerns about health and addiction were the two most reported reasons across products, whereas economic reasons were the third most reported reason among those attempting to quit cigarettes and smokeless nicotine. Focusing on some of these reasons in future interventions or counseling settings may be useful in preventing the use of nicotine products among youth. Additionally, product-specific reasons to quit might be useful for counselors struggling to guide and maintain youth using non-cigarette products. Still, the findings should be examined by longitudinal studies, to fully understand the causality and mechanisms that motivate youth to quit and succeed.

## Supplementary Material



## Data Availability

The data supporting this research are available from the authors on reasonable request.
