# Supplementary material for: Reasons to quit cigarettes, e-cigarettes, and smokeless nicotine products: A cross-sectional study among Danish people aged 15–29 years
Source: Tob Prev Cessat. 2026 May 22;12:10.18332/tpc/215586. doi: 10.18332/tpc/215586 (PMC13197859; doi:10.18332/tpc/215586)
Supplement: Supplementary file 1 [file TPC-12-25-s1.pdf]

Supplementary Table 1. Odds ratios of reporting the five most frequent reasons to quit across product used. Unadjusted, and stratified by quit success, Denmark, 2023, (N=2948[b]).

	**Thoughts about my Health**			**Don't want to be addicted**			**Due to economics**			**Switched to another product**			**People I know privately**		
	OR	95% CI	P-value	OR	95% CI	P-value	OR	95% CI	P-value	OR	95% CI	P-value	OR	95% CI	P-value
Product type			**<0.001[e]**			**<0.001[e]**			**<0.001[e]**			**<0.001[e]**			**<0.001[e]**
Cigarette	*Reference*			*Reference*			*Reference*			*Reference*			*Reference*		
E-cigarette	0.60	(0.50; 0.72)	**<0.001**	0.79	(0.65; 0.95)	**0.014**	0.50	(0.39; 0.63)	**<0.001**	1.16	(0.92; 1.47)	0.207	0.77	(0.61; 0.98)	**0.030**
Smokeless nicotine	1.49	(1.26; 1.77)	**<0.001**	2.36	(1.99; 2.81)	**<0.001**	1.68	(1.41; 2.01)	**<0.001**	0.20	(0.13; 0.29)	**<0.001**	1.31	(1.07; 1.60)	**0.008**
**Stratified by quit success**		*P = 0.173[a]*			*P = 0.539[a]*			*P = 0.857[a]*			*P = 0.712[a]*			***P = 0.022[a]***	
	OR	95% CI	P-value	OR	95% CI	P-value	OR	95% CI	P-value	OR	95% CI	P-value	OR	95% CI	P-value
**Unsuccessful** (N=1367[c])															
Product type			**<0.001[e]**			**<0.001[e]**			**<0.001[e]**			**<0.001[e]**			**0.036[e]**
Cigarette	*Reference*			*Reference*			*Reference*			*Reference*			*Reference*		
E-cigarette	0.84	(0.57; 1.21)	0.344	0.96	(0.65; 1.41)	0.840	0.56	(0.36; 0.86)	**0.008**	0.77	(0.41; 1.47)	0.432	1.38	(0.89; 2.12)	0.149
Smokeless nicotine	1.56	(1.23; 1.97)	**<0.001**	2.45	(1.94; 3.10)	**<0.001**	1.47	(1.17; 1.85)	**0.001**	0.19	(0.10; 0.37)	**<0.001**	1.39	(1.07; 1.81)	**0.015**
**Successful** (N=1581[d])															
Product type			**<0.001[e]**			**<0.001[e]**			**<0.001[e]**			**<0.001[e]**			**0.011[e]**
Cigarette	*Reference*			*Reference*			*Reference*			*Reference*			*Reference*		
E-cigarette	0.55	(0.44; 0.69)	**<0.001**	0.77	(0.61; 0.97)	**0.029**	0.65	(0.48; 0.87)	**0.004**	0.92	(0.72; 1.20)	0.568	0.66	(0.49; 0.88)	**0.006**
Smokeless nicotine	1.36	(1.03; 1.78)	**0.028**	2.11	(1.60; 2.78)	**<0.001**	1.51	(1.11; 2.05)	**0.009**	0.25	(0.16; 0.41)	**<0.001**	1.08	(0.77; 1.53)	0.647

Bold: Significance ($p<0.05$); [a]P-value for overall interaction between product type and quit success on the reason to quit, [b]2948 observations corresponding to 2228 individuals; [c]1367 observations corresponding to 1230 individuals; [d]1581 observations corresponding to 1305 individuals, [e]P-value for overall difference between products. Abbreviations: CI, Confidence Interval.

© 2026 Arp S. et al.
